# Role of wild birds in the circulation of *Toxoplasma gondii* in Southern Italy: molecular and epidemiological insights

**DOI:** 10.3389/fvets.2025.1745864

**Published:** 2026-01-29

**Authors:** Francesco Serra, Stefano Scarcelli, Giovanni Sgroi, Marita Georgia Riccardi, Milad Badri, Marco Paone, Simona Rea, Martina Levante, Emanuela Di Lecce, Giorgia Borriello, Bianca Cecere, Nicola D'Alessio, Vincenzo Veneziano, Giorgio Galiero, Orlando Paciello, Giuseppe Iovane, Maria Grazia Amoroso

**Affiliations:** 1 Department of Animal Health, Experimental Zooprophylactic Institute of Southern Italy, Naples, Italy; 2 Department of Veterinary Medicine, University of Naples Federico II, Naples, Italy; 3 Department of Sciences and Technologies, University of Sannio, Benevento, Italy; 4 Medical Microbiology Research Center, Qazvin University of Medical Sciences, Qazvin, Iran; 5 Osservatorio Faunistico Venatorio–Campania Region, Naples, Italy

**Keywords:** Apicomplexa, bradyzoites, oocysts, *Pica pica*, real-time PCR

## Abstract

**Introduction:**

Toxoplasmosis is caused by the protozoal parasite *Toxoplasma gondii* and can be acquired through the consumption of food and water contaminated with sporulated oocysts and tissue cysts. The parasite is characterized by high host flexibility, being able to infect all warm-blooded animals, including birds and mammals. Wild birds are considered important reservoirs of infectious agents, some of which possess zoonotic potential. To date, few data are available on the role of these animals in the transmission of *T. gondii*, especially in the Italian Peninsula.

**Methods:**

To address this gap, the present study investigated the circulation and genetic diversity of this parasite in various wild bird species, with the aim to assess the role of avian hosts in the epidemiology of the parasite. In 2023–2024, 256 carcasses belonging to 39 different wild bird species were collected in Campania and Calabria Regions and analyzed to assess the presence of *T. gondii* in the animal tissues.

**Results:**

Out of 256 birds, 15 (5.9%) resulted positive for parasite DNA: 6 (40.0%) belonged to genotype GII and 1 (6.6%) to genotype GIII. Interestingly, 11/15 (73.3%) infected animals were non-migratory species and 10/15 (66.7%) were classified as omnivore/scavenger wild birds.

**Discussion:**

Monitoring and genotyping *T. gondii* in birds could help to understand the environmental spread of oocysts. What's more, given the remarkable ecological diversity of wild avian species (different feeding habits and migratory behavior), surveillance of avian populations could deepen our understanding of *T. gondii* transmission dynamics and implement public health interventions and environmental management strategies.

## 1 Introduction

*Toxoplasma gondii* (phylum Apicomplexa, family Sarcocystidae) is an obligatory intracellular protozoan causing toxoplasmosis (1). The parasite can infect a wide group of warm-blooded animals (including humans) inducing a widespread infectious disease with medical and veterinary importance worldwide (2–4). In Europe, prevalence varies between species and regions being also influenced by rearing (indoor/outdoor) (5). Higher prevalences were found in Eastern Europe compared to Northern Europe and in outdoor-kept animals (5). In small ruminants, especially goats and sheep, there is a widespread distribution of *T. gondii*, reaching seroprevalence values of 93.7%−98.4% in a study conducted in Spain (6). In these animals, the parasite causes reproductive disorders, abortion, congenital malformation and stillborn (6–8). The life cycle of *T. gondii* is characterized by sexual reproduction, involving definitive hosts such as cats and other felids, and asexual reproduction, occurring in intermediate hosts such as mammals and birds (9, 10). The parasite had been previously classified into three predominant lineages, known as types I, II and III. Type I strains are highly virulent, whereas type II and type III are relatively nonvirulent (11). Subsequently, through new genotyping techniques and the use of a greater number of genetic markers, several new and atypical genotypes have been identified (12, 13). However, archetypal haplotypes, identified as genotypes I, II and III still play a central role in genotyping as they provide a stable phylogenetic reference for placing new isolates (14, 15). Clonal haplotypes, moreover, have biological and clinical value, as they are associated with differences in virulence and pathogenicity, and serve as reference markers to identify any recombination or atypical lineage (15, 16). Genotypes I and III, in particular, are widespread in Europe and North America. This is likely due to the combination of specific alleles during recombination, providing a selective advantage to these genotypes (17). *T. gondii* infection can occur through ingestion of water and vegetables contaminated with sporulated oocysts, unpasteurised milk containing tachyzoites, or raw/undercooked meat with pseudocysts (18). The consumption of fresh meat and meat products indeed represents an important source of human infection (19). Food and Agriculture Organization of the United Nations (FAO) classified toxoplasmosis among the 10 most important foodborne diseases of the world (20). In humans, toxoplasmosis is asymptomatic in immunocompetent people but can cause severe and fatal symptoms in immunosuppressed patients and in the fetus of pregnant women (9, 21). A high circulation and prevalence of *T. gondii* in intermediate hosts, both farmed animals and wildlife, has already been widely documented in Southern Italy (20, 22–24). In a study conducted between 2020 and 2022 in Campania region, *T. gondii* was detected in various wild mammal species with a non-negligible overall prevalence of 21.8% (*n* = 46/211) (20). In another study *T. gondii* DNA was found in the tissues of 78/177 (44%) wild boars examined (23). The seroprevalence of the parasite in livestock has been evaluated in a study conducted on sheep farms, in which 91 out of 117 (77.8%) farms tested had at least one animal IgG positive (15). In another study carried out on water buffaloes, 17 out of 124 (13.7%) animals tested positive for *T. gondii* IgG (17). To date, limited information is available on the potential role of wild birds in the spread of *T. gondii* infection. These animals due to their high dispersal capability and high ecological plasticity, are important reservoirs of infectious agents and play a crucial role in the spread and transmission of pathogens, some of which possess zoonotic potential (25–28). Indeed, these animals are frequently involved in the transmission cycles of various viruses, such as West Nile virus (26) and avian influenza viruses (25), as well as of parasites (27, 28). Birds may therefore represent a group at considerable risk for *T. gondii* infection and could act as a key link in the parasite's transmission, given their wide ecological distribution, diverse feeding habits, and role as prey for multiple predators (29). With respect to feeding habits, recent molecular studies interestingly showed that insect physiology can vary considerably (30, 31), and these variations may influence ecological interactions between insects and their predators, including insectivorous birds, potentially affecting the birds' exposure to *T. gondii*. Additionally, the availability and abundance of insect prey can strongly modulate these interactions, as insectivorous birds rely on fluctuations in insect populations for feeding and reproductive success (32). The coexistence of migratory and resident species further enhances their epidemiological relevance: while migratory birds can disseminate the parasite over long distances, potentially introducing *T. gondii* into previously uninfected areas, resident species may function as indicators of local environmental contamination, reflecting ongoing transmission dynamics within a specific ecosystem (29, 33–35). To date, few molecular studies were conducted on *T. gondii* infection in wild birds especially in Southern Italy. Furthermore, knowledge about the circulating genotypes and ecological host factors is limited. Therefore, the aim of the present study was to investigate, the presence of *Toxoplasma gondii* in wild birds collected in Southern Italy. Further genotyping was carried out with the scope to characterize the circulating parasite variants. In addition, the likely influence of feeding habits and migratory behavior of wild birds on *T. gondii* infection was investigated, considering the hypothesis that parasite exposure could be somehow influenced by birds' lifestyle.

## 2 Materials and methods

### 2.1 Study area and sampling

No approval from ethical committee was required, as all sampling procedures were run under the frame of a regional plan for wildlife surveillance (authorization no. DD 210-B7 DPAR), according to EU and National legislations. A total of 256 wild bird carcasses were collected between January 2023 and December 2024, from provinces of Campania (228) and Calabria (28) regions (Southern Italy). The two regions extend from 0 to 1,890 meters above sea level and are mainly characterized by hilly landscape and typical Mediterranean climate, with dry summers and rainy winters (22). All the carcasses (except crows) were found on the road following citizens' sighting and collected by veterinary practitioners involved in the regional plan; the crows came instead from regular hunting activities. All the carcasses were delivered at the Istituto Zooprofilattico Sperimentale del Mezzogiorno (Italy) for a complete necropsy examination, according to the regional plan for wildlife surveillance. For each animal, brain, heart, and skeletal muscle samples were collected with sterile scalpels, placed in sterile tubes, and stored at−20 °C for subsequent molecular investigations. Data on each bird's sex and species was recorded, and age was determined based on plumage (36). All the carcasses were identified at species level through the evaluation of anatomical features assessed by a veterinary practitioner specialized in avian pathology. In detail, 247 birds were adults (96.5%) and 9 juveniles (3.5%). Among the 256 specimens, 129 were males (51.4%) and 127 were females (49.6%). Furthermore, information on bird principal feeding habits was recorded. The 256 wild birds belonged to 39 species, of which 168 (65.6%) were non-migratory and 88 (34.3%) were migratory (Table 1).

**Table 1:** Wildlife avian species investigated (39 in total).

**Bird species**	**Migratory**	**Principal feeding habit**
Blackbird (*n* =2) *Turdus merula*	Yes	Omnivore
Cattle egret (*n* = 2) *Bubulcus ibis*	Yes	Insectivore
Common buzzard (*n* = 6) *Buteo buteo*	Yes	Carnivore
Common kestrel (*n* = 2) *Falco tinnunculus*	Yes	Carnivore
Common raven (*n* = 1) *Corvus corax*	No	Omnivore/scavenger
Common starling (*n* = 2) *Sturnus vulgaris*	Yes	Omnivore
Crow (*n* = 86) *Corvus corone*	No	Omnivore/scavenger
Duck (*n* = 1) *Anas* spp.	Yes	Omnivore
Eurasian golden oriole (*n* = 1) *Oriolus oriolus*	Yes	Insectivore/frugivore
Eurasian jay (*n* = 18) *Garrulus glandarius*	Yes	Omnivore/granivore
Eurasian magpie (*n* = 58) *Pica pica*	No	Omnivore/scavenger
Eurasian sparrowhawk (*n* = 2) *Accipiter nisus*	Yes	Carnivore
Eurasian skylark (*n* = 1) *Alauda arvensis*	Yes	Insectivore/granivore
Eurasian teal (*n* = 2) *Anas crecca*	Yes	Omnivore
European turtle dove (*n* = 5) *Streptopelia turtur*	Yes	Granivore
Eurasian woodcock (*n* = 3) *Scolopax rusticola*	Yes	Insectivore
Great crested grebe (*n* = 2) *Podiceps cristatus*	Yes	Piscivore
Gull (*n* = 1) *Larus* spp.	Yes	Omnivore/scavenger
Herring gull (*n* = 9) *Larus michahellis*	Yes	Omnivore/scavenger
Hooded crow (*n* = 9) *Corvus cornix*	No	Omnivore/scavenger
House sparrow (*n* = 1) *Passer domesticus*	Yes	Granivore
Little owl (*n* = 4) *Athene noctua*	Yes	Carnivore
Mallard (*n* = 3) *Anas platyrhynchos*	Yes	Omnivore
Mediterranean gull (*n* = 1) *Larus melanocephalus*	Yes	Omnivore/scavenger
Muscovy duck (*n* = 4) *Cairina moschata*	No	Omnivore
Northern goshawk (*n* = 2) *Accipiter gentilis*	Yes	Carnivore
Northern lapwing (*n* = 1) *Vanellus vanellus*	Yes	Insectivore
Osprey (*n* = 1) *Pandion haliaetus*	Yes	Piscivore
Peregrine falcon (*n* = 3) *Falco peregrinus*	Yes	Carnivore
Red kite (*n* = 2) *Milvus milvus*	Yes	Carnivore/scavenger
Rock dove (*n* = 8) Columba livia	No	Granivore
Song thrush (*n* = 5) *Turdus philomelos*	Yes	Omnivore
Swan (*n* = 1) Cygnus spp.	Yes	Herbivore
Tawny owl (*n* = 1) *Strix aluco*	No	Carnivore
Water rail (*n* = 2) *Rallus aquaticus*	Yes	Omnivore
Woodpecker (*n* = 1) *Pucumnus* spp.	Yes	Insectivore
Western barn owl (*n*=1) *Tyto alba*	Yes	Carnivore
Western jackdaw (*n* = 1) *Coloeus monedula*	Yes	Omnivore/scavenger
White stork (*n* = 1) Ciconia ciconia	Yes	Carnivore
Total (*n* = 256)	**–**	**–**

Animals were classified as migratory/non-migratory and also with respect to their principal feeding habit. n, number of animals analyzed for each species.

### 2.2 Nucleic acids extraction procedures

Preliminary experiments were carried out with the aim of establishing the most efficient extraction procedure. For the scope, different sample quantities (25, 50 and 100 mg) were homogenized with phosphate-buffered saline (PBS) solution or ATL solution (Qiagen GmbH, Hilden, Germany). Prior to extraction a pretreatment stage of the homogenate (500 μl) with 20 μl of a 20 mg/ml proteinase K solution (Qiagen GmbH, Hilden, Germany) was also carried out. For the scope, the sample was incubated at two different temperatures (56 or 70 °C) for 20 min prior to extraction. The best results were obtained with 25 mg of sample homogenized in PBS by Tissue Lyser (Qiagen GmbH, Hilden, Germany) and directly (without pretreatment) extracted. All the successive experiments were therefore performed following preliminary results. Accordingly, each sample was inserted in a 2 ml Eppendorf safe-lock tube containing 1 ml PBS and a 4.8-mm stainless steel bead. Mechanical lysis was carried out at 30 Hz for 5 min; this step was followed by sample centrifugation at 2,000–4,000 rpm for 15 min to pellet the debris. Nucleic acids were extracted from 200 μl homogenate using QIAsymphony automated extraction system (Qiagen GmbH, Hilden, Germany) with the DSP Virus/Pathogen Mini kit (Qiagen GmbH, Hilden, Germany), according to the manufacturer's instructions and eluted in 80 μl elution buffer. A sample made with 200 μl PBS was used as a negative process control (NPC). Furthermore, an external positive process control (EPC), murine norovirus, was spiked into each sample (including NPC) prior to extraction, and used to evaluate PCR inhibitors (37). Murine norovirus amplification results were interpreted as follows: if the threshold cycle (Ct) of the EPC in the eluted sample was comparable to that of the EPC in the NPC, the sample was further analyzed as undiluted. If, instead, the difference between the two Cts was at least three or a multiple of three, all the analyses were carried out on the sample diluted 1:10 or more (considering one decimal dilution every three threshold cycles of difference).

### 2.3 Real-time PCR for detection of *Toxoplasma gondii*

Molecular detection of *T. gondii* was carried out by a protocol identifying a small fragment of the B1 gene. The reaction was performed on a QuantStudio five Real-Time PCR thermal cycler (Thermo Fisher Scientific, Waltham, Massachusetts, USA) in a total volume of 25 μl containing 5 μl DNA extracted, 12.5 μl Universal Master Mix PCR 1X (Thermo Fisher Scientific, Waltham, Massachusetts, USA), 1 μl (12.5 μM) forward primer (TOXO-For 5′- TCCCCTCTGCTGGCGAAAACT 3′), 1 μl (12.5 μM) reverse primer (TOXO-Rev 5′-AGCGTTCGTGGTCAACTATCGATTG3′) and 0.5 μl (10 μM) probe TOXO-P (FAM5′-TCTGTGCAACTTTGGTGTATTCGCAG3′-TAMRA) (23). The thermal profile used was the following: initial denaturation at 95 °C for 15 min, 45 cycles of 95 °C for 15 s and 60 °C for 60 s.

### 2.4 Genotyping analysis of *Toxoplasma gondii*

Genetic characterization of *T. gondii* in positive samples was performed by Multiple Locus Variable Number of Short Tandem Repeats analysis (MLVA), based on the amplification of five microsatellite (MS) markers (TUB2, W35, TgM-A, B18 and B17) by multiplex PCR assay (38). This technique, is indeed more sensitive than PCR-RFLP and shows a higher level of resolution in detecting genetic diversity among genetically closely related *T. gondii* isolates (14, 16, 39, 40). Furthermore, STR analysis is faster than multilocus sequencing studies, which are time-consuming and unsuitable for a large set of isolates (38). In the end, STR typing represents the current reference method for *T. gondii* molecular characterization, thus allowing comparisons between strains collected from different geographical areas or animal species (41). PCR was performed in a 25 μl reaction mixture consisting of 12.5 μl of 2X Qiagen Multiplex PCR Master Mix (Qiagen, Hilden, Germany), 5 μl of Q solution 1X and 0.04 μM of each primer. Five microliters of the DNA from the positive samples were added to the reaction mixture. DNA from ATCC strains, PTG strain Type II (ATCC: 50941), CTG Type III (ATCC: 50842) and MAS atypical strain (ATCC: 50870), already genotyped previously (42) were used as positive controls. In each PCR reaction a negative control was added, represented by Rnase/Dnase free water (NC). Amplification was carried out in a SimpliAmp Touch thermal cycler (ThermoFisher Scientific, Waltham, MA, USA) and consisted of an initial denaturation at 95 °C for 15 min followed by 45 cycles consisting of 94 °C for 30 s, 55 °C for 3 min and 72 °C for 60 s. The last extension step was carried out at 60 °C for 30 min. One microliter of PCR product was mixed with 0.3 μl of LIZ 500 Standard Size and 13.7 μl of HIDi Formamide. The mixture was then denatured for 5 min at 95 °C and resolved by capillary electrophoresis with an ABI PRISM 3500 genetic analyzer (ThermoFisher Scientific, Waltham, MA, USA), equipped with a 50 cm long capillary filled with POP-7 separation medium. Analysis of the microsatellite fragments was carried out using GeneMapper software v5.0. The minimum fluorescence threshold for valid peaks was set at 200 RFU.

### 2.5 Statistical analysis

Exact binomial 95% confidence intervals (95% CIs) by Wilson's method were calculated for the proportions of infection herein found. A chi-square test (χ^2^) was used to assess any statistical difference in the frequency of infection, based on bird's species (migratory/non-migratory), age (juvenile/adult), sex, geographical origin and feeding habits. Considering the different diets of the wild birds, a statistical analysis was carried out grouping the species in two categories based on their principal feeding habits: “meat consumers” which included: omnivore, carnivore, piscivore, omnivore/scavenger, carnivore/scavenger, omnivore/granivore and non-meat consumers including: insectivore, herbivore, granivore, insectivore/granivore, insectivore/frugivore. The value of *p* < 0.05 was considered statistically significant. The Odds ratio (*OR*) was used to verify difference of infection risk according to the geographical origin of birds. Statistical analyses were performed by using the online software Epitools-Epidemiological Calculators (43). The distribution of *T. gondii*-positive birds in the study area was obtained with QGIS software (version 3.34.10-Prizren LTR).

## 3 Results

**Figure 1:**
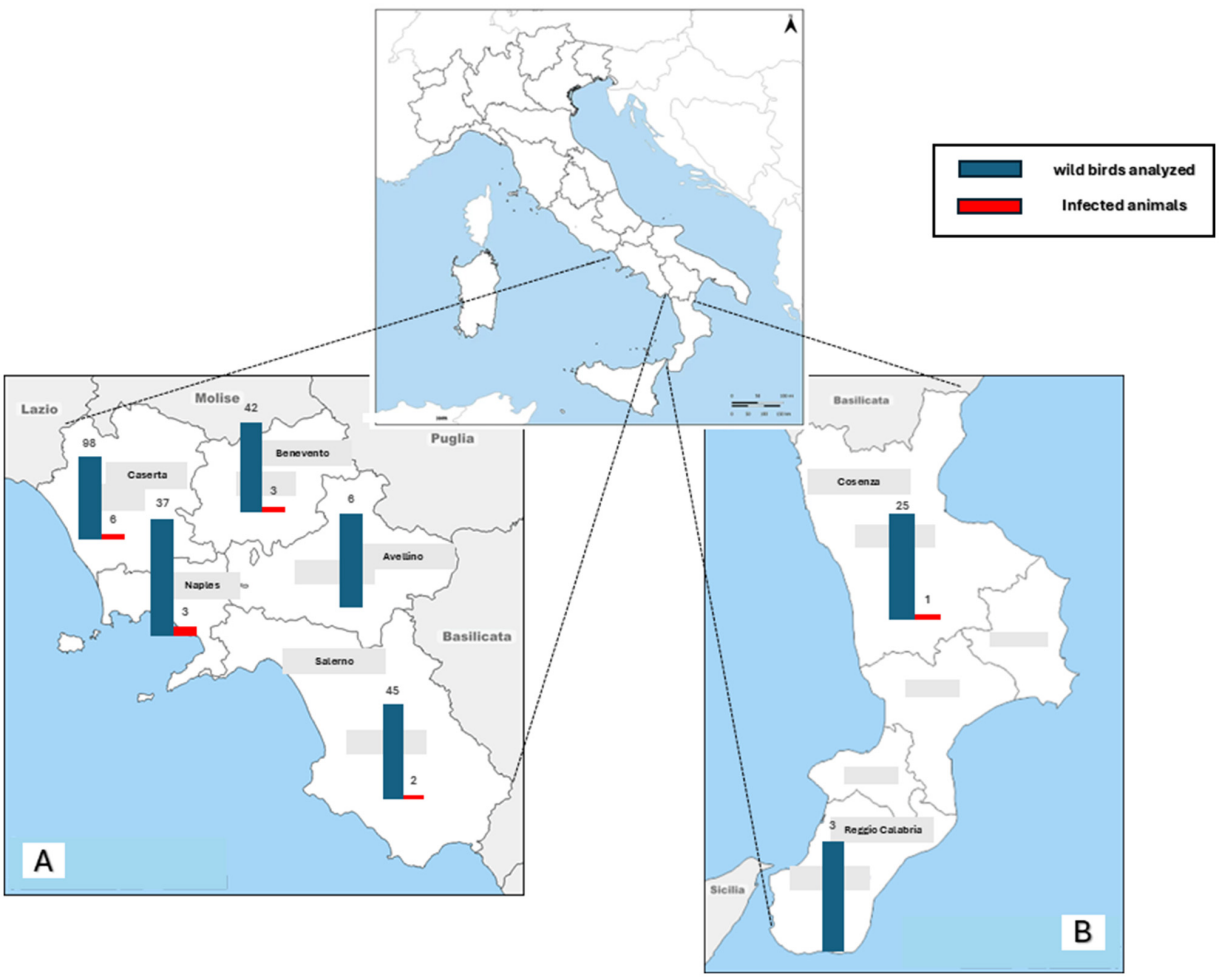
*Toxoplasma gondii*-positive birds in Campania **(A)** and Calabria **(B)** regions, southern Italy, in 2023–2024.

Out of 256 bird specimens analyzed, 15 (i.e., 5.9%, 95% CI: 3.6–9.4) resulted positive for *T. gondii* DNA, with Ct values ranging between 32.5 and 37. Parasite DNA was detected in the heart and muscle of 9 infected animals and in the brain of five birds. Furthermore, it was found in more than one tissue in seven of the 15 infected animals (46.6%; see Table 2). Parasite genotyping, carried out by MLVA characterization, assigned 6/15 (40%) *T. gondii* as belonging to GII genotype, 1/15 (6.6%) to GIII genotype. In eight animals, *T. gondii* was not characterized probably due to the poor quality or quantity of the genetic material. As shown in Figure 1, positive birds were distributed across the study area, with 14/15 (93.3%) animals from Campania Region and 1/15 (6.7%) from Calabria Region. With respect to species, *T. gondii* was found in 6/58 *Pica pica* (10.3%), 4/86 *Corvus corone* (4.6%), 1/4 *Cairina moschata* (25.0%), 1/4 *Athene noctua* (25.0%), 1/2 *Falco tinnunculus* (50.0%), 1/18 *Garrulus glandarius* (5.5%) and 1/2 *Podiceps cristatus* (50.0%; Table 3). The 15 infected birds were all adults, eight of which males (53.3%) and seven females (46.7%). Interestingly, most of the positive birds (11/15, 73.3%) belonged to non-migratory species. Furthermore, when looking at their feeding habits, all were meat-consumers and 10/15 (66.7%) were omnivore/scavengers. Statistical analyses, reported in Table 4, revealed no significant difference in *T. gondii* prevalence with respect to migratory behavior (*p* = 0.520), principal feeding habits (*p* = 0.167), sex (*p* = 0.450), age (*p* = 0.450), and geographical origin (*p* = 0.970). No significant difference (*p* = 0.187) was also observed when comparing parasite prevalence in the two wild bird species most frequently collected (*C. corone, n* = *86*; *P. pica, n* = *58)*. A higher infection risk was however found in adult birds compared to juvenile ones (*OR* = 5.8) and in “meat consumers” compared to the “non-meat consumers” (*OR* = 6.4).

**Table 2:** Prevalence of *Toxoplasma gondii* in the tissues investigated (brain, heart, and skeletal muscle; *n* = 256).

**Sample**	**Pos (%)**	**95% CI**
Brain	2 (0.8)	0.2–2.8
Heart	3 (1.2)	0.4–3.4
Skeletal muscle	3 (1.2)	0.4–3.4
Subtotal	8 (3.1)	1.6–6.0
**Coinfections**			
Brain-heart	1 (0.4)	< 0.001–2.2
Brain-skeletal muscle	1 (0.4)	< 0.001–2.2
Heart-skeletal muscle	4 (1.6)	0.6–3.9
Brain-heart-skeletal muscle	1 (0.4)	< 0.001–2.2
Subtotal	7 (2.7)	1.3–5.5
Total	15 (5.9)	3.6–9.4

Pos/Tot (%), number and percentage of T. gondii-positive samples; CI, 95% confidence interval.

**Table 3:** Wild birds in which *T*. *gondii* DNA was identified.

**Bird**	**Bird species**	**Sex**	**Age**	**Migratory**	**Principal feeding habits**	***T. gondii* genotype**
1	Common kestrel (*Falco tinnunculus*)	Male	Adult	Yes	Carnivore	GII
2	Crow (*Corvus corone*)	Female	Adult	No	Omnivore/scavenger	Not characterized
3	Crow (*Corvus corone*)	Female	Adult	No	Omnivore/scavenger	Not characterized
4	Crow (*Corvus corone*)	Male	Adult	No	Omnivore/scavenger	GIII
5	Crow (*Corvus corone*)	Female	Adult	No	Omnivore/scavenger	Not characterized
6	Eurasian magpie (*Pica pica*)	Male	Adult	No	Omnivore/scavenger	GII
7	Eurasian magpie (*Pica pica*)	Female	Adult	No	Omnivore/scavenger	GII
8	Eurasian magpie (*Pica pica*)	Female	Adult	No	Omnivore/scavenger	Not characterized
9	Eurasian magpie (*Pica pica*)	Female	Adult	No	Omnivore/scavenger	Not characterized
10	Eurasian magpie (*Pica pica*)	Male	Adult	No	Omnivore/scavenger	Not characterized
11	Eurasian magpie (*Pica pica*)	Male	Adult	No	Omnivore/scavenger	Not characterized
12	Eurasian jay (*Garrulus glandarius*)	Male	Adult	Yes	Omnivore/carnivore	GII
13	Great crested grebe (*Podiceps cristatus*)	Male	Adult	Yes	Piscivore	GII
14	Little owl (*Athene noctua*)	Male	Adult	Yes	Carnivore	GII
15	Muscovy duck (*Cairina moschata*)	Female	Adult	No	Omnivore	Not characterized

All these species were classified as “meat consumers.” Twelve species belong to Corvidae family. (Not corvids are: Common kestrel, Great crested grebe and Muscovy duck).

**Table 4:** Prevalence of *Toxoplasma gondii* according to migratory behavior, principal feeding habits, sex, age, and geographic origin.

**Variables**	**Pos/Tot (%)**	**95% CI**	***χ2*; *p***	***OR***
**Migratory behavior**				
Migratory	4/88 (4.5)	1.8–11.1	0.42; 0.520	0.7
Non-migratory	11/168 (6.5)	3.7–11.3		
**Principal feeding habits**				
Meat consumers	15/215 (17.8)	4.1–11.2	1.90; 0.167	6.4
Non-meat consumers	0/41 (0)	0.0–10.2		
**Sex**				
Male	8/129 (6.2)	3.2–11.8	0.05; 0.810	1.1
Female	7/127 (5.5)	2.7–10.9		
**Age**				
Juvenile	0/9 (0)	–	0.58; 0.450	5.8
Adult	15/247 (6.1)	3.7–9.8		
**Province**				
Avellino	0/6 (0)	–	1.36; 0.970	–
Benevento	3/42 (7.1)	2.5–19.0		
Caserta	6/98 (6.1)	2.8–12.7		
Cosenza	1/25 (4.0)	0.7–19.5		
Napoli	3/37 (8.1)	2.8–21.3		
Reggio Calabria	0/3 (0)	–		
Salerno	2/45 (4.4)	1.2–14.8		

Pos/Tot (%), Number and percentage of T. gondii-positive birds out of the total examined; 95% CI, 95% confidence interval; χ2, p, chi-square and p-value; OR, odds ratio.

## 4 Discussion

Our study focused on the detection of *T. gondii* in wild birds from the provinces of the Campania and Calabria regions (Southern Italy). Molecular investigations revealed a non- negligible positivity rate of 5.9%, which is, however, lower than those reported in the few previous studies on avian species carried out in Italy, particularly in Central and Northern regions (33, 44, 45). An epidemiological investigation performed on wild birds from Central Italy reported serological and molecular prevalences of 11.6% (*n* = 25/216) and 8.8% (*n* = 19/216), respectively (33). In a recent study carried out in 2021–2022, a positivity rate of 14% was found on wild waterbirds (44). This difference in molecular prevalence could be related to the species analyzed (39 in our study) as well as to the uneven distribution of cysts in the animal tissues which may determine a casual underestimation of parasite prevalence (46). Higher prevalence rates of *T. gondii* have been documented in wild bird species more likely to be exposed to infection due to their feeding habits and ecological behavior. For instance, an epidemiological study on wild birds of prey hospitalized in Wildlife Recovery Center (WRCs) in Northern Italy, showed a positivity rate of 62.5% (45). As a matter of fact, raptors can become infected through predation on birds and small mammals, thereby harboring tissue cysts and acting as intermediate hosts within the complex life cycle of the parasite (47–49). In the present study, *T. gondii*, investigated in 39 wild bird species, was detected with a slightly higher prevalence (6.5%) in non-migratory birds with respect to migratory ones (4.5%), although statistical analysis revealed no significant difference. Migratory species may represent an important potential vector for the spread of infection to new geographic areas, whereas non-migratory ones represent a sentinel of the local environmental contamination. Moreover, non-migratory species living in human-dominated habitats are exposed to higher infection risk due to the elevated density of free-roaming domestic cats and consequent soil contamination (50–53). Recent habitat-ecology studies suggested that urban and anthropogenic landscapes can provide suitable habitats for certain bird species, shaping community composition and favoring species that tolerate human-modified environments (54), thereby increasing their likelihood of encountering contaminated resources. Wild birds investigated, when grouped according to their primary feeding habits, revealed a higher infection risk (*OR* = 6.4) in the “meat consumers” group compared with the “non-meat consumers.” Interestingly, all 15 infected animals belonged to the “meat consumers” group, and twelve of them belonged to the Corvidae family. Corvids are very versatile and opportunistic in their feeding behavior, acting as scavenger that consume animal carcasses, exploit anthropogenic food waste and prey on live animals (55–57). The susceptibility of these non-migratory birds to *T. gondii* infection has already been reported in the literature. In an epidemiological survey of 771 wild corvids, (651 magpies and 120 hooded crows *Corvus cornix*), hunted for faunistic restoration across various areas in Central Italy, 45 birds (41 magpies and four hooded crows) were found positive for anti-*T. gondii* antibodies (5.8%). Therefore, carnivorous and scavenger species appear to be at higher risk of *T. gondii* infection, likely influenced by dietary habits (45, 58). In line with Wilson et al. (59), these findings emphasize that feeding ecology is a key determinant of *T. gondii* exposure risk in wild birds. Our results showed that the parasite was found with interesting prevalence of 10.3% in Eurasian magpies and of 4.6% in crows. Terrestrial carnivores and omnivores, such as magpies and crows, acquire infection through consumption of tissue cysts present in infected vertebrates, whereas herbivores and insectivores are mainly exposed via oocysts in soil, water, or invertebrates (60, 61). Furthermore, opportunistic carnivores with scavenging behavior, (such as magpies and crows, exploit carcasses, anthropogenic food waste, and live prey), thereby increasing the likelihood of exposure to *T. gondii* (58, 62, 63). Other investigated variables, such as sex and age did not show any statistically significant effect on parasite prevalence, consistent with previous findings (58, 64). Nevertheless, a higher infection risk (*OR* = 5.8) was observed in adult birds compared to juvenile specimens. This aligns with another study on pet birds, which reported a significantly higher prevalence of *T. gondii* in adult animals (13.2%) than in juveniles (5.3%; OR = 2.76; *p* = 0.004) (65).

Regarding *T. gondii* genetic diversity, MLVA analysis revealed, in the infected birds, the presence of two distinct genotypes: type II (six samples) and type III (one sample). These results are consistent with previous studies reporting the circulating *T. gondii* genotypes in Europe, where type II and, to a lesser extent, type III strains, dominate in both domestic and wild environments (66–68). The advent of Whole Genome Sequencing (WGS) and single nucleotide polymorphisms (SNPs) analysis improved our knowledge of the genotypic structure and the evolutionary history of *T. gondii*. Minot et al. (17) used WGS to analyze isolates belonging to major clonal genotypes and atypical strains, mapping thousands of SNPs distributed across the genome and detecting recombination events, genomic mosaics and tissue tropism differences with high resolution. More recent studies, including Joeres et al. (41), further consolidated the use of genome-wide SNPs panels for *T. gondii* genotyping, applying them to clinical samples and field isolates. Analysis of SNPs allows for more precise definition of phylogenetic relationships between strains and identification of recombinant lineages. However, the routine use of WGS and SNPs analysis remains limited due to high costs, bioinformatics complexity, and the need for high DNA quantities and qualities, often unavailable in clinical samples. Therefore, MLVA analysis still represents the current reference standard for *T. gondii* genotyping (41), particularly in epidemiological, surveillance and inter-laboratory comparison studies, being a good compromise between discriminating ability, speed, reproducibility and cost, allowing the distinction of closely related isolates even from low amounts of DNA. Interestingly, *T. gondii* DNA was detected in all analyzed organs, most frequently in the heart and skeletal muscle, consistent with the parasite's known tissue tropism in wild birds (33). The presence of tissue cysts in edible muscles supports the potential for transmission via meat consumption. Wild birds can be therefore a source of infection for felids and other animals that consume many domestic and wild birds as part of their diet (12). As to humans, in this study, *T. gondii* was found in seven different wild bird species of which, only one is enters human food diet. Interestingly, *T. gondii* was indeed detected in one of the four *Cairina moschata* specimens analyzed. Muscovy ducks are non-migratory wild birds whose meat is highly appreciated in several countries for its nutritional qualities (69). Moreover, these birds are valued for their relatively higher breast and leg meat yield, and their lower content of skin and abdominal fat. Therefore, the detection of *T. gondii* in *Cairina moschata* is particularly relevant, as this species may enter human food chains, highlighting a potential public health risk associated with the consumption of its meat. Indeed, the consumption of their raw or undercooked meat is considered an important source of human infection in various countries (12, 70–72). According to the literature, there is no scientific evidence of human consumption of meat from the other infected species. Poor palatability, legal restrictions, and religious beliefs may contribute to their limited consumption. Indeed, some wild birds, such as raptors and corvids, are considered mythical, spiritual, and religious symbols in many cultures (73, 74). In addition, these animals are protected by European law (Birds Directive 2009/147/EC), which prohibits their hunting, killing, or consumption (75, 76).

Overall, the present study gives interesting results on *T. gondii* prevalence and distribution in poorly investigated but with high reservoir potential animals like wild birds. Important bias must be however taken into account: for some species the sample size was small compared to others like corvids. This was due to the fact that animals were collected following a Regional plan (found dead or hunted) and have not been selected upstream. The different sample size of the species investigated thus reflects their different abundance in Campania and Calabria Region (77, 78). Furthermore, due to the challenges encountered during molecular investigations and the poor quality of the genetic material, *T. gondii* was not characterized in all animals analyzed. A limitation of the present survey, which is also a future perspective, is the focus only toward *T. gondii* instead of other zoonotic foodborne parasites potentially present in birds, such as *Trichinella* spp. and *Sarcocystis* spp., that have been reported to circulate in wild mammals (boars, wolves, foxes) in the same study area (79, 80).

## 5 Conclusions

In summary, this study highlights the importance of monitoring *Toxoplasma gondii* and its genotypes in wild birds and shows that the risk of *T. gondii* infection is likely influenced by these animals' dietary habits. The detection of the parasite in a species consumed by humans further emphasizes the critical role of surveillance within a One Health framework. This integrated perspective is fundamental for the development and implementation of effective strategies to control the spread of *T. gondii* as well as other zoonotic foodborne parasites.

## Data Availability

The original contributions presented in the study are included in the article/supplementary material, further inquiries can be directed to the corresponding author/s.
